# Using patient data to optimize an expert-based guideline on convalescence recommendations after gynecological surgery: a prospective cohort study

**DOI:** 10.1186/s12893-017-0317-8

**Published:** 2017-12-06

**Authors:** Esther V. A. Bouwsma, Johannes R. Anema, A. Vonk Noordegraaf, Henrica C. W. de Vet, Judith A. F. Huirne

**Affiliations:** 1 Department of Obstetrics and Gynecology, VU University Medical Center, P.O. Box 7057, Amsterdam, 1007 MB The Netherlands; 2 Department of Public and Occupational Health, VU University Medical Center, Amsterdam, The Netherlands; 3 EMGO Institute for Health and Care Research, Amsterdam, The Netherlands; 4 Department of General Practice, VU University Medical Center, Amsterdam, The Netherlands; 5 Department of Epidemiology & Biostatistics, VU University Medical Center, Amsterdam, The Netherlands

**Keywords:** Convalescence advice, return to normal activities, return to work, hysterectomy, laparoscopic adnexal surgery, eHealth

## Abstract

**Background:**

Convalescence advice is often based on tradition and anecdote from health care providers, rather than being based on experiences from patients themselves. The aim of this study was to analyse recovery in terms of resumption of various daily activities including work, following different laparoscopic and abdominal surgery in order to optimize an expert-based guideline on convalescence recommendations.

**Methods:**

This is a prospective cohort study conducted in nine general and one university hospital in the Netherlands. Women aged 18–65 years and scheduled for a hysterectomy (laparoscopic, vaginal, abdominal) and/or laparoscopic adnexal surgery (*n* = 304) were eligible to participate. Preoperatively, participants were provided with tailored expert-based convalescence recommendations on the graded resumption of several daily activities including sitting, standing, walking, climbing stairs, bending, lifting, driving, cycling, household chores, sport activities and return to work (RTW). Postoperatively, time until the resumption of these activities was tracked. Convalescence recommendations were considered correct when at least 25% and less than 50% of the women were able to resume an activity before or at the recommended recovery time.

**Results:**

There was a wide variation in the duration until the resumption of daily activities within and between groups of patients undergoing different types of surgery. Recovery times lengthened with increasing levels of physical burden as well as with increasing levels of invasiveness of the surgery. For the majority of activities actual recovery times exceeded the recovery time recommended by the expert panel.

**Conclusions:**

This study provided insight in the resumption of daily activities after gynecological surgery and the adequacy of an expert-based convalescence guideline in clinical practice. Patient data was used to optimize the convalescence recommendations.

**Trial registration:**

Dutch trial registry, NTR2087 (August 2009) and NTR2933 (June 2011).

**Electronic supplementary material:**

The online version of this article (10.1186/s12893-017-0317-8) contains supplementary material, which is available to authorized users.

## Background

The importance of perioperative education to prepare patients for the postoperative period has been topic of research for decades [1–5]. It has been demonstrated that perioperative education can increase patient satisfaction, reduce pain as well as psychological distress and can optimize patients’ expectations [6–10]. Notwithstanding, evidence based perioperative education has not yet found its way into routine surgical care [11–14]. Mainly two reasons can be identified for this. First, there is only little evidence on the duration needed to resume various daily activities following different surgeries [15–20]. This leads to convalescence advise being based on tradition and anecdote from health care providers, rather than being based on experiences from patients themselves [14, 15, 18, 21–24]. Second, due to the current trend towards day care and short stay surgery, patient contact is very brief and time available for patient education has practically evaporated [25–29].

In order to optimize perioperative care in the Netherlands, our research group developed an expert-based multidisciplinary guideline on convalescence recommendations following four types of gynecological surgery. Using a structural consensus method, an expert panel of gynecologists, general practitioners and occupational physicians formulated recommended recovery times for the graded resumption of 38 daily activities (e.g. standing, walking, climbing stairs, performing household chores, and return to work (RTW)) [30, 31]. These convalescence recommendations were then incorporated in a web-based care program. The effect of this intervention care program on duration of sick leave was evaluated rigorously [32–35].

The objective of the current study were twofold. First, we wanted to use the collected patient data in order to describe the resumption of daily activities, including return to work, following four types of gynecological surgery in patients who were exposed to the expert-based convalescence recommendations. Second, we intended to use this patient data to optimize the expert-based convalescence guideline in pursuance of increasing the evidence on convalescence recommendations.

## Methods

This prospective cohort study was carried out with data collected in two consecutive multicenter trials studying the effectiveness of a multidisciplinary care program aimed at improving recovery and preventing delayed return to work following benign gynecological surgery. Details of the study designs, as well as the results of the efficacy, process evaluation, effectiveness and cost-effectiveness studies have been published previously [32–37].

### Study population

All women aged between 18 and 65 years, employed for at least 8 h per week (salary employed, self-employed, or voluntary work), and scheduled for a surgery for benign disease in one of the participating ten hospitals were eligible to participate. The types of surgeries that were included were: laparoscopic adnexal surgery (LAS) and/or laparoscopic hysterectomy (LH), vaginal hysterectomy (VH) or abdominal hysterectomy (AH). Patients with severe comorbidity – described as major health problems affecting daily activities or recovery – were excluded, as the intervention was developed for *healthy* patients undergoing *uncomplicated* surgical procedures. Patients were also excluded if they were diagnosed with a malignancy, were pregnant, were computer or Internet illiterate, were involved in a lawsuit against their employer, were on disability sick leave before surgery, or had insufficient command of Dutch.

This study was performed with the participants randomized to the intervention group, because only they received structured convalescence recommendations. Participants that filled in the web-based recovery monitor on the web portal at least twice formed the study population, as they were the participants that provided data on the resumption of their daily activities.

### Intervention

**Fig. 1:**
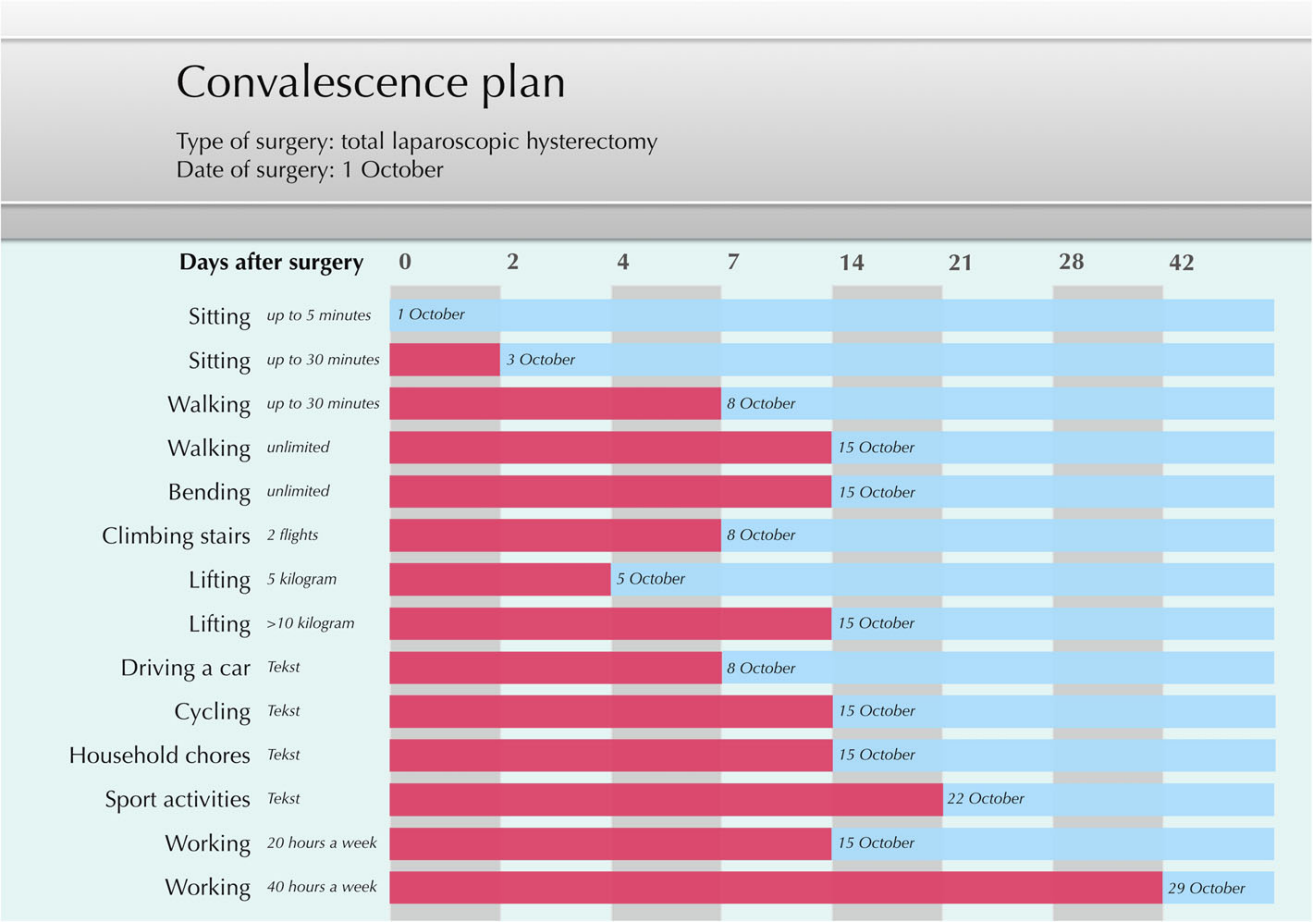
Example of a tailored convalescence plan generated at the patient web portal. In the left column activities are listed that were selected by the patient. The pink boxes present the amount of time the patient is recommended to avoid the specific activity. The blue boxes present the duration after surgery (and the specific date) after which the patient is recommended to resume the specific activity

The intervention program was comparable in both trials. Patients in the intervention group received access to a patient web portal on which they were encouraged to generate a personalized convalescence plan. This convalescence plan included tailored recommendations for the graded resumption of daily activities based on an algorithm of the expert-based guideline on convalescence recommendations. Figure 1 illustrates an example of a tailored convalescence plan generated at the patient web portal.

### Outcomes

The expert-based convalescence guideline included recommended recovery times for 38 activities. For the current study the following ten daily activities were selected: sitting, standing, walking, climbing stairs, bending, lifting, driving, cycling, performing household chores, and performing sport activities. These activities were considered as most common and essential for daily living (Additional file 1). In addition, they showed a wide variation in physical burden as well. The first six activities consisted of different grades of ability, i.e. different recommended recovery times were given for the partial resumption of that activity. To illustrate, the activities sitting, standing, and walking were graded for different durations (e.g. 15 min, 30 min or more than 60 min). The activities climbing stairs, bending and lifting were graded in number of flights, degrees, and weight, respectively.

Lastly, the outcome time to full sustainable RTW was also included in the current study, defined as the resumption of own work or other work with equal earnings, for at least 4 weeks without (partial or full) recurrence.

### Data collection

Socio-demographic data were collected by a baseline questionnaire. A web-based recovery monitor on the patient web portal was used to collect data about the duration until the resumption of daily activities. At 2, 4, 7, 14, 28, 56, and 84 days (=12 weeks) after surgery participants were asked to track the activities that they were able to perform and the activities that were still experiencing problems with (e.g. riding a bike, performing household chores). Graded activities were tracked separately. For example, for the activity lifting, participants were asked whether they were able to lift 5 kg (yes/no), 10 kg (yes/no) and 15 kg (yes/no). Once a certain activity could be performed without problems, this activity was removed from the recovery monitor. Completion of the recovery monitor was not obligatory, as a result duration of follow-up could vary. Patients were also allowed to complete additional reports between the set time points.

Sick leave data were collected by monthly, self-reported sick leave calendars during the six months after surgery. In addition, duration until RTW was also tracked with the web-based recovery monitor.

### Data analysis

Excel 2010 (Microsoft, Washington, DC, USA) was used to transform the weblog into user statistics. SPSS version 22.0 (IBM Corporaton, Amonk, NY, USA) was used for descriptive and statistical analyses.

Due to user authentication (username and password), website activity was logged for each individual participant and it was therefore possible to determine the date at which a recovery monitor was filled in. All data entries were used, except monitors that were filled in retrospectively (later than the next set time point).

To investigate the role of missing data, baseline characteristics and duration until full sustainable RTW were compared between participants that filled in the web-based recovery monitor and the participants that did not, using independent t-test and Pearson’s Chi –squared test for continuous and categorical variables respectively. Subgroups were formed by patients that underwent different types of surgery, to analyze the relation between the level of invasiveness of a procedure and the length of recovery.

Time until the resumption of daily activities was determined by calculating the mean between the first time point at which a certain activity could be performed and the last time point at which that activity could not be performed. To illustrate, when a patient reported at 14 days she could not ride a bike and she reported she was able to do this at day 28, the mean recovery time was calculated to be 21 days. For graded activities the resumption of the different gradations was calculated separately in the same manner. Recovery times were truncated to integer numbers. Times until the resumption of normal activities were analyzed by means of descriptive statistics using the median and interquartile range (IQR) for each activity in each procedure. Boxplots were used to present the data graphically.

Duration of sick leave was determined by calculating the time difference between the surgery and the date of full sustainable RTW. Duration of sick leave were depicted graphically for each type of surgery using the Kaplan-Meier method. To analyze differences in RTW between the different surgical types the log rank test was used.

For each activity, the percentage of patients was determined that was able to perform that activity before or at the recommended recovery time. The expert-based convalescence recommendations were considered correct when at least 25% of the population was able to resume an activity before or at the recommended recovery time. The 25th percentile was selected as a cut-off because it was hypothesized that convalescence recommendations should motivate patients to resume their daily activities, yet should not be too challenging resulting in discouragement. In addition, the chosen cut-off also takes into account that there might be some delay between the recommended recovery time and the actual resumption of a certain activity under real life circumstances. To illustrate, we hypothesized that when less than 25% of the participants were able to perform an activity before or at the recommended recovery time, the expert-bases convalescence advise was too strenuous. Similarly, when more than 50% of the participants were able to perform an activity before the recommended recovery time, the expert-based convalescence recommendation was considered as too tolerant.

Patient data were then used to revise the convalescence guideline in case recommended recovery times were too strenuous or too tolerant. This process included two steps. First, the recovery time at the 25th percentile was calculated per (graded) activity for each type of surgery. As the expert panel formulating the original guideline used a fixed schedule of time points (1 – 2 – 4 – 7 – 10 – 14 – 21 – 28 – 42 days following surgery) we used the same mutation moments for the revision of the guidelines. In other words, when the 25th percentile was calculated at 4 days, the revised recommended recovery time would be 4 days. However, when the 25th percentile was calculated at 5 days, the revised recommended recovery time would become 7 days. During the second step, the revised guidelines were compared between the different surgery types. When actual recovery times for the same activity in a more invasive surgery group exceeded the revised recommended recovery times, the revision was undone.

## Results

The first randomized study ran from March 2010 until September 2011 and of the 215 patients, 110 patients were allocated to the intervention group. The second trial ran from October 2011 until July 2014 and of the 433 patients, 227 patients were included in the intervention group. Thus, in total 337 patients were exposed to the expert-based convalescence recommendations and were eligible for data analysis for this current study. In total, 304 of these 337 patients (90.2%) completed the recovery monitor at least twice and they formed the study population of this study (Additional file 2).

For the resumption of daily activities, the median length of follow-up was 12 weeks (IQR: 6–12 weeks) and on average, participants filled in the recovery monitor seven times (IQR: 4–8). The median number of days between two data registrations was 9 (IQR: 7–12). Length of follow-up for the outcome RTW was 182 days. Table 1 presents the baseline characteristics of the study population. The majority of patients were in their forties, were intermediate or highly educated and were salary-employed. Baseline characteristics did not differ between participants undergoing different types of surgery nor between participants that filled in the web-based recovery monitors and those that did not.

**Table 1:** Baseline characteristics (*N* = 304)

**Patient characteristics**	
Age (years ± SD)	45.3 ± 7.5
Dutch nationality	292 (96.1%)
Education level^a^	
Low	33 (10.9%)
Intermediate	124 (40.8%)
High	147 (48.4%)
Smoking status	
None-smoker	176 (57.9%)
Former-smoker	66 (21.7%)
Current-smoker	62 (20.4%)
**Surgery-related characteristics**	
Type of surgery	
Laparoscopic adnexal surgery	109 (35.9%)
Laparoscopic hysterectomy	79 (26.0%)
Vaginal hysterectomy	58 (19.1%)
Abdominal hysterectomy	58 (19.1%)
**Health-related characteristics**	
Perceived health status (median (IQR))	80.0 (70.0–90.0)
Under treatment by another specialist	130 (42.8%)
History of previous abdominal surgery	110 (36.2%)
**Work-related characteristics**	
Type of work	
Salary employed	256 (84.2%)
Self-employed	42 (13.8%)
Voluntary work	6 (2.0%)
Work hours per week (mean ± SD)	29.9 ± 9.4
Sick leave prior to surgery^b^	108 (35.5%)
RTW expectation (long)^c^	50 (16.4%)
RTW intention (low)^d^	66 (21.7%)

Data present the number of patients (%), unless otherwise indicated

^a^Low = preschool, primary school; intermediate = secondary school; high = tertiary school, university, or postgraduate

^b^Defined as at least 1 day of abcence

^c^Defined as expectation longer than 3 weeks for adnexal surgery, longer than 6 weeks for laparoscopic or vaginal hysterectomy, or longer than 8 weeks for abdominal hysterectomy

^d^A higher score indicates a higher intention to return to work despite physical symptoms (range 1–5). A low intention was defined as score 1 or 2

### Return to normal activities

The percentage of patients that were able to perform the daily activities before or at the time of the recommended recovery time varied between 4 and 78% depending on the activity as well as the type of surgery (Table 2). The recommendations for VH fitted reality the best (13 correct recommendations and only one too strenuous) followed by the recommendations for AH (ten correct and two too strenuous). The recommendations for LAS were too strenuous for half of the activities.

**Table 2:**
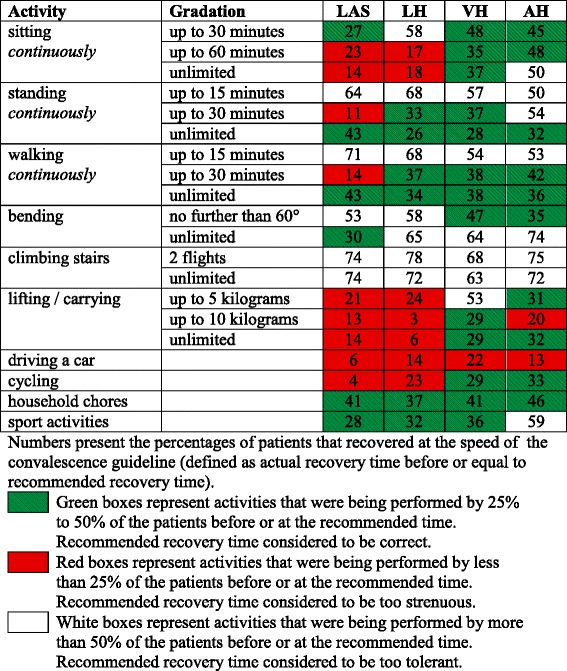
Percentages of patients recovering slower, equal, or faster than recommended

The activities standing (15 min), walking (15 min) and climbing stairs were performed by more than 50% of the participant across all surgical types before or at the recommended time. The recommended recovery times for the activities sitting, lifting and cycling were determined correctly for the surgery types VH and AH, however, they were too strenuous for patients undergoing LAS and LH. Across all surgical types, participants resumed driving much later than recommended.

**Fig. 2:**
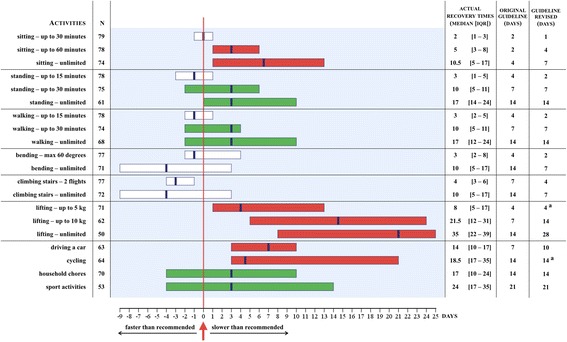
Differences between actual and recommended recovery times after laparoscopic hysterectomy. The vertical red line presents the convalescence guideline. The boxplots present the 25th percentile, median (thick vertical line) and 75th percentile of the differences between actual recovery times and the recommended recovery times. White boxes present activities that were being performed by more than 50% of the patients before or at the recommended recovery time. Green boxes present activities that were being performed by 25% to 50% of the patients faster than the recommended recovery times. Red boxed present activities that were being performed by less than 25% of the patients faster than the recommended recovery times.^a^ guidelines were not revised due to algorithm taking other surgical types into account (lifting 5 kg, cycling). N, number of patients that provided data on the activity; IQR, interquartile range

Figure 2 shows the difference between actual and recommended recovery times to the (partial) resumption of several daily activities following LH. It also demonstrates how the guideline was revised for the activities for which the recommended recovery times were too strenuous or too tolerant.

**Fig. 3:**
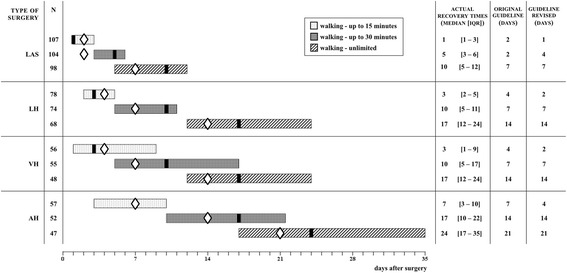
Actual and recommended recovery times for the activity walking per type of surgery. The boxplots present the 25th percentile, median (thick vertical line) and 75th percentile of the actual recovery times. The diamonds represent the recommended recovery times. LAS, laparoscopic adnexal surgery; LH, laparoscopic hysterectomy; VH, vaginal hysterectomy; AH, abdominal hysterectomy

Figure 3 shows the actual and recommended recovery times for the graded activity walking across the four types of surgeries. Conform the recommended recovery times formulated by the expert panel, recovery times became longer with each gradation, as well as with higher levels of invasiveness of the surgical procedure. Notably, accuracy decreased with longer recovery times, demonstrated by the increasing interquartile ranges.

### Return to work

**Fig. 4:**
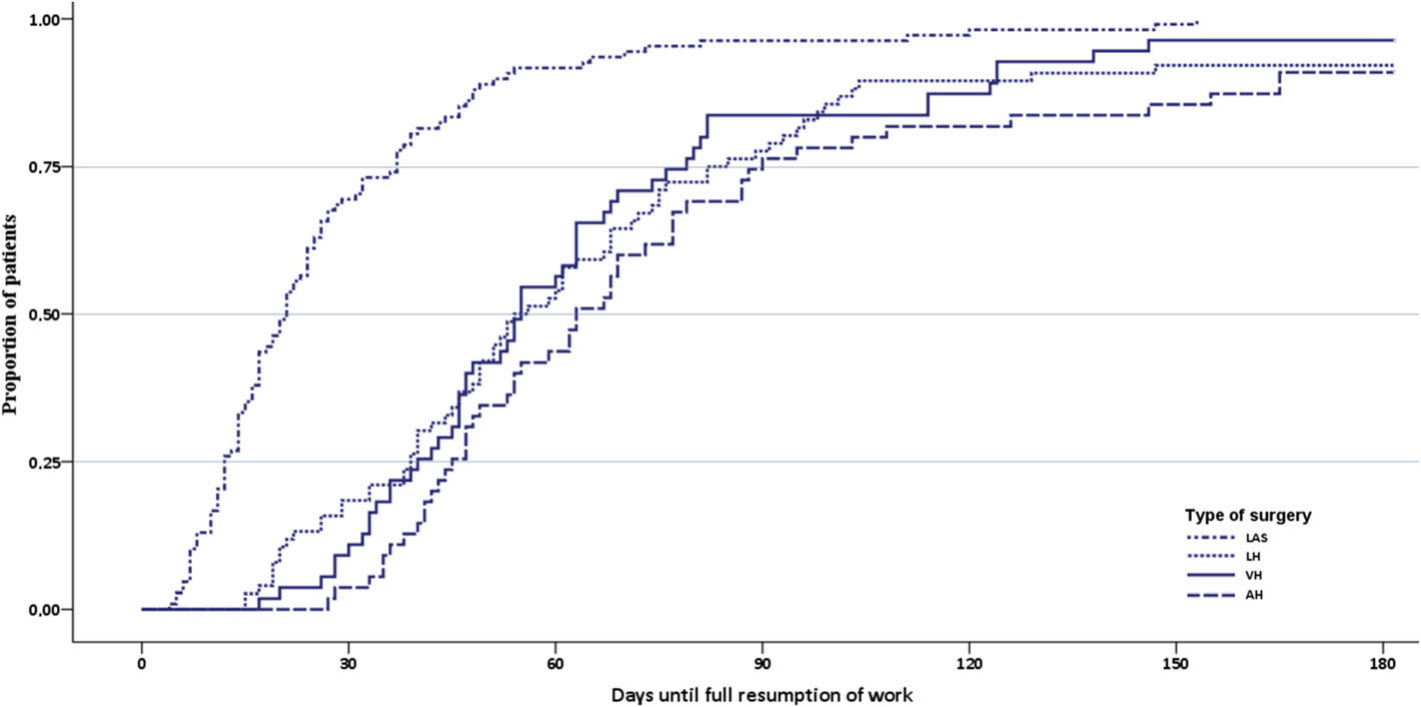
Kaplan-Meier survival curves for time to full sustainable RTW, presented per type of surgery. Number of days represent days of sick leave after surgery until RTW

Median times to RTW were 21 days for LAS (95% CI: 17.7–24.3), 56 days for LH (95% CI: 47.4–64.7), 55 days for VH (95% CI: 46.8–63.2), and 68 days for AH (95% CI: 62.1–73.9). Thirteen patients were censored at 182 days because they were still on sick leave. Duration until full sustainable RTW following the four surgical types differed significantly (log rank test: *P* < 0.000) (Fig. 4).

Actual times to RTW were longer than the recommended times for most of the gradations in the work categories (Table 3). Recommended recovery times for the least invasive surgery group (LAS) and the most invasive group (AH) were closer to the actual recovery times than the recommended recovery times for the intermediate invasive surgery group (LH and VH). There was no difference in duration until RTW between the patients included in this study and those that were excluded because they did not complete the web-based recovery monitor at least twice.

**Table 3:** Actual recovery times for the (graded) resumption of work

Type of surgery (N)	20 h per week		30 h per week		40 h per week	
	n	Median (IQR)	n	Median (IQR)	n	Median (IQR)
LAS (109)	87	8 (5–15)	77	16 (9–24)	61	18 (12–33)
LH (79)	62	27 (14–35)	45	35 (19.5–49)	32	39 (24–51.3)
VH (58)	37	35 (23.5–46)	30	38 (35–49)	20	49 (39.8–52)
AH (58)	40	35 (24–49)	30	40 (32.3–60.8)	24	50 (35–60.5)

Data present the median number of days after surgery at which the activity could be performed. N: number of patients per surgery group; n: number of patients that provided data on the activity

### Complicated surgeries

In total, 19 patients experienced a complication, defined as a significant larger surgery than planned or a repeat surgery related to the initial surgery: 5 patients in the LAS group (4.6%), 3 patients in the LH group (3.8%), 3 patients in the VH group (5.2%), and 8 patients in the AH group (13.8%). To investigate if this group influenced the recovery rates, we repeated the analyses excluding those patients with a complicated procedure. Surprisingly, this did not lead to significantly better recovery rates. Instead, for some activities the recovery rates became poorer, indicating that a complicated procedure does not necessarily means prolonged recovery.

## Discussion

### Main findings

In this study we used prospectively collected data about the time until the resumption of ten daily activities as well as the duration until full sustainable work following four types of gynecological surgeries in order to describe median recovery times. In addition, the collected patient data enabled us to optimize an earlier developed expert-based guideline on convalescence recommendations following gynecological surgery for benign disease, and revise recommended recovery times if they turned out to be too strenuous or too tolerant. For the majority of activities actual recovery times exceeded the recovery time recommended by the expert panel. Yet, recovery times lengthened with increasing levels of physical burden of the daily activities as well as with increasing levels of invasiveness of the procedures, conform the algorithm of the expert-based convalescence guideline. The convalescence guideline seemed more accurate for patients undergoing more complex surgery than patients undergoing minimal invasive surgeries, as the recommendations in the latter group were often too strenuous.

### Data interpretation

Several survey studies conducted in the last two decades inventorying convalescence recommendations following gynecological procedures demonstrated that there is substantial variation in convalescence advice given by health care providers and emphasized the need for unified convalescence guidelines [11, 12, 16, 24, 38, 39]. However, we are not aware of research similar to our own, in which both input from experts as well as input from patients were used to generate convalescence recommendations. The ultimate goal of our research is to develop a set of general convalescence recommendations that is applicable to the majority of patients undergoing several types of gynecological surgery.

The current study can be used as an example to build the evidence base for convalescence recommendations in the surgical field. Mainly, there are three reasons why this should be on top of the agenda of policy makers. First of all, the availability of evidence-based guidelines will facilitate care providers to provide their patients with more specified and tailored advice [14]. Secondly, it has been previously demonstrated that standardized convalescence recommendations can expedite recovery [33, 34, 40–43]. Thirdly, a more standardized post-operative trajectory would also allow the identification of patients who deviate from the norm and prompt the possibility of intervention [20, 25].

In our study, we observed a wide variation in the duration until the resumption of daily activities within groups of patients undergoing the same surgical procedure. In a *post-hoc* analysis we investigated a number of potential determining factors for delayed recovery. The results were not straightforward, and therefore, difficult to interpret. For example, for several activities, we found a significant association between the level of education and the length of recovery (lower education leading to longer recovery). Possibly, education is a proxy for the type of work a patient is performing (sedentary work versus manual labor), however, with the available data we were not able to investigate this relationship any further. The age of the patient did not seem to be an independent factor for delayed recovery. Understanding these mechanisms in the future, would probably help to identify those patients that need more guidance or monitoring during their recovery.

### Strengths and limitations

Several strengths of the present study are notable. First of all, data about the resumption of daily activities was collected prospectively, reducing the risk of recall bias. Secondly, we used a relative long follow-up period (12 weeks for daily activities and 26 weeks for RTW) and from a medical point of view it generally may be assumed that the daily activities should have been resumed within this time period. In our study, the vast majority of patients achieved full RTW within 26 weeks (96.1%). In addition, we focused on both the resumption of daily activities as well as RTW. The selected daily activities had a wide variation of physical burden and RTW was considered as the most demanding activity, as it generally requires performing a whole set of single activities. Therefore, RTW is an outcome that is frequently used to define the end of the surgical recovery process [44]. Another strength of the current study is that advice given to patients was standardized as patients were provided with tailored convalescence recommendations based on the expert-based guideline. In this way, other factors that might influence recovery, such as patient expectations and contradictive advise, were reduced [10, 45].

Our study also has limitations. Regarding methodology, bias may have been introduced because the web-based recovery monitor was not obligatory to complete. This could have led to both over- and underestimations of recovery times, as patients who did not use the web-based recovery monitor could have been the fast recoverees (no need to use the web portal anymore), or the slow recoverees (discouraged by the web portal, and therefore avoiding it). As sick leave duration did not differ significantly between patients who did and who did not use the recovery monitor, we expect the effect of this type of selection bias to be minimal in our study.

Secondly, we collected recovery data by asking patients to track the activities they were able to perform at given set time points prospectively, instead of asking the exact date at which the participant resumed that particular activity. Therefore, we were obliged to estimate at what moment the mutation took place, which we did by calculating the mean between the first time point at which a certain activity could be performed and the last time point at which that activity could not be performed. As the length between set time points increased (the frequency of data-collection decreased), the estimates became less accurate, demonstrated by the wide IQRs for the activities with relative high physical burden. Unfortunately, this phenomenon of decreasing accuracy with time was amplified, due to increasing numbers of patients lost to follow-up with time.

### Practical and research implications

As stated before, future research should focus on identifying predictors of recovery. Moreover, the relationship between recommended and actual recovery times should be investigated, especially focusing on the question if there is a turning point at which too strenuous recommendations can become preposterous and will lead to delayed recovery. In addition, it should be examined which factors (emotional or physical) determine if a patient will comply to convalescence recommendations given. Future challenges will also involve the dissemination, adaptation and implementation of the convalescence guidelines in daily practice. It should be noted that recovery outcomes may be different across populations due to differences at the level of the health care systems as well as cultural diversity, making external generalization of our guideline uncertain [44].

Ultimately, convalescence advice should be tailored to the individual patient, also taking into account other patient characteristics such as age and the presence of any co-morbidity, as well as environmental factors such as specific job demands. Hypothesizing, when detailed recovery data were to be centrally registered, advanced data methods (i.e. big data) could be applied to predict personal recovery and generate custom-made convalescence recommendations for surgical patients on a wider scale [46]. In this perspective, smart wearables can be useful for monitoring postoperative physical activity as a proxy of recovery, and simultaneously providing the input for such predictive models [47–50].

## Conclusions

We described recovery times of various daily activities including work, following four types of gynecological surgeries. Collected patient data were used to revise a previously developed expert-based guideline on convalescence recommendations. This study should be considered as an important step towards the development of evidence-based convalescence advice, leading to the optimization of perioperative gynecological care. Future research should focus on the adaptation of these convalescence recommendations and its implementation into routine surgical care.

## Additional files

Additional file 1: Overview of activities included in the developed convalescence guideline. (DOCX 16 kb)

Additional file 2: Organization of the cohort. (DOCX 31 kb)
