# Supplementary material for: Using patient data to optimize an expert-based guideline on convalescence recommendations after gynecological surgery: a prospective cohort study
Source: BMC Surg. 2017 Dec 6;17:129. doi: 10.1186/s12893-017-0317-8 (PMC5719670; doi:10.1186/s12893-017-0317-8)
Supplement: Supplementary file 1 — Overview of activities included in the developed convalescence guideline. (DOCX 16 kb) [file 12893_2017_317_MOESM1_ESM.docx]

**Online Resource 1.** Overview of activities included in the developed convalescence guideline

| **Category** | **Dichotomous activities ^a^** | **Graded activities (unit) ^b^** |
| --- | --- | --- |
| Personal functioning | memory | concentration (duration) |
|  | insight | dividing attention (duration) |
|  | action tempo |  |
| Static and dynamic movements | reaching | **continuous sitting (duration)** |
|  | handling above shoulder height | total sitting (duration per day) |
|  | handling heavy objects | **continuous standing (duration)** |
|  | kneeling / squatting | total standing (duration per day) |
|  | continuous kneeling / squatting | **continuous walking (duration)** |
|  | twisting upper body | total walking (duration per day) |
|  | continuous bending / twisting | **bending (angles)** |
|  |  | bending frequently (duration) |
|  |  | reaching frequently (duration) |
|  |  | **lifting / carrying (weight)** |
|  |  | pushing / pulling (weight) |
|  |  | handling light objects (duration) |
|  |  | **climbing stairs (number of flights)** |
|  |  | climbing a ladder (height) |
| Working |  | **hours per day (duration)** |
|  |  | **hours per week (duration)** |
|  |  | performing shift work (day, evening, night) |
| Other activities | **household chores  ^c^** |  |
|  | **performing sport activities ^d^** |  |
|  | bathing |  |
|  | sexual intercourse |  |
|  | **cycling** |  |
|  | **driving** |  |
|  | commuting |  |
| **Bold activities were selected for the current paper.**  ^a^ Dichotomous categories have two options: able to perform versus not able to perform (or impaired).  ^b^ Graded activities are divided in different gradations using different units (e.g. the activity ‘lifting’ is divided into: up to 5, up to 10, and up to 15 kilograms and the activity “continuous waking” is divided into: up to 15, up to 30, and more than 30 minutes.)  ^c^ Any activity comparable to vacuum cleaning.  ^d^ Any activity comparable to jumping. | | |
