# Supplementary figures and images for: Using patient data to optimize an expert-based guideline on convalescence recommendations after gynecological surgery: a prospective cohort study
Source: BMC Surg. 2017 Dec 6;17:129. doi: 10.1186/s12893-017-0317-8 (PMC5719670; doi:10.1186/s12893-017-0317-8)

**Online Resource 2.** Organization of the cohort


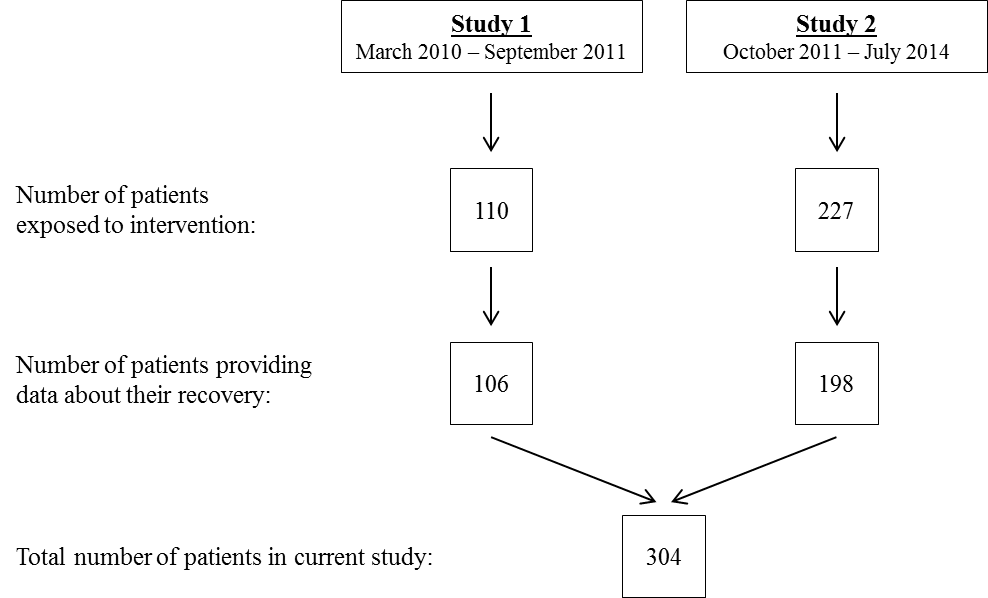

Supplement: Supplementary file 2 — Organization of the cohort. (DOCX 31 kb) [file 12893_2017_317_MOESM2_ESM.docx]
